# EZH2–STAT3 signaling pathway regulates GSDMD-mediated pyroptosis in glioblastoma

**DOI:** 10.1038/s41420-024-02105-0

**Published:** 2024-07-28

**Authors:** Dong Yu, Shuai Wang, Jiajun Wang, Kang Zhang, Zihui Niu, Ning Lin

**Affiliations:** Department of Neurosurgery, The affiliated Chuzhou Hospital of Anhui Medical University, The First People’s Hospital of Chuzhou, Chuzhou, Anhui China

**Keywords:** CNS cancer, Inflammasome, Necroptosis

## Abstract

Glioblastoma multiforme (GBM) is the most therapeutically challenging primary brain tumor owing to the unique physiological structure of the blood–brain barrier. Lately, research on targeted therapy for gliomas has shifted focus toward the tumor microenvironment and local immune responses. Pyroptosis is a newly identified cellular demise characterized by the release of numerous inflammatory factors. While pyroptosis shows promise in impeding the occurrence and progression of GBM, the regulatory mechanisms governing this process in gliomas still require further investigation. The function of the Enhancer of zeste homolog 2 (EZH2) in pyroptosis remains unexplored. In this study, we discovered that 3-Deazaneplanocin A (DZNep), an inhibitor of EZH2, can induce pyroptosis in GBM in vitro experiments. Moreover, our investigation unveiled that the signal transducer and activator of transcription (STAT3) could serve as a downstream regulator influenced by EZH2, impacting pyroptosis in GBM. Following treatment with DZNep and the STAT3 inhibitor (SH-4–54), there was an elevation in the levels of pyroptosis-related factors, namely NOD-like receptor thermal protein domain-associated protein 3 (NLRP3) and Gasdermin D (GSDMD). Moreover, simultaneous inhibition of both EZH2 and STAT3 led to the expression of inflammatory factors such as IL-1β and IL-18. In summary, we have identified that EZH2 regulates pyroptosis in GBM through STAT3, and pyroptosis could potentially be targeted for immunotherapy in GBM.

## Introduction

Characterized by rapid growth, strong invasiveness, and high fatality rates, glioblastoma multiforme (GBM) stands as the most prevalent primary brain tumor [1]. Current treatments for GBM primarily involve surgical intervention, supplemented by radiotherapy and chemotherapy [2]. However, due to the unique tissue structure of GBM, with unclear boundaries between tumor and normal tissues, the recurrence rate after surgery remains high. GBM patients have a median survival rate of only 15 months [3]. Additionally, the efficacy of chemotherapy for GBM is suboptimal, the efficacy of GBM treatment is compromised by the presence of the blood–brain barrier [4]. Current research focuses mainly on unraveling the mechanisms of GBM, with inducing programmed cell death in GBM being a major research direction [5, 6]. Studies on apoptosis and autophagy in GBM are relatively mature, with several validated molecular pathways [7, 8]. However, research on pyroptosis in GBM is still in the experimental stage [9], lacking relevant molecular mechanisms. Understanding the molecular signaling pathways regulating pyroptosis in GBM could provide potential therapeutic directions for glioma patients.

Pyroptosis, reliant on the gasdermin protein family, is a regulated form of cell death, resulting in membrane perforation [10]. Pyroptotic cells undergo swelling occurs until membrane rupture happens, leading to the efflux of intracellular factors. During this process, there is often a significant release of pro-inflammatory cytokines, including IL-1β and IL-18, thereby triggering a robust inflammatory response [11]. In the classical pathway of cell pyroptosis, Caspase-1 cleaves Gasdermin D (GSDMD), yielding cleaved N-terminal GSDMD (N-GSDMD). As a result, membrane pores form, potassium effluxes, and numerous inflammatory factors are released into the extracellular space. Ultimately, these events cause cell death and alter the tumor microenvironment [12, 13]. Furthermore, pyroptosis activation requires the NOD-like receptor family pyrin domain-containing 3 (NLRP3) inflammasome, a pivotal molecule for assembling the inflammasome [14, 15]. Only after the inflammasome binds to Caspase-1 can GSDMD be cleaved, inducing cell pyroptosis [16]. Recent studies generally consider that cell pyroptosis plays a role in various cellular physiological processes, particularly in the growth of malignant tumors, as it can directly lyse cells and induce immune cell infiltration, suppressing tumor growth [17, 18].

EZH2 often plays a role as a cancer promoter in gliomas, primarily regulating gene expression at the epigenetic level by catalyzing trimethylation of lysine 27 on histone H3 (H3K27me3) [19, 20]. Studies have examined the role of EZH2 in apoptosis, autophagy, and cell cycle regulation in gliomas [21, 22]. Additionally, studies have shown that the EZH2 inhibitor DZNep can cross the blood–brain barrier and exert effects on central nervous system research [23, 24]. However, its role in pyroptosis is currently unclear. Furthermore, EZH2 serves as an upstream regulator for various signaling factors, including the signal transducer and activator of transcription 3 (STAT3). In the journal Cancer Cell, Eunhee Kim et al. demonstrated that in glioblastoma (GBM), EZH2 binds to and methylates STAT3, which enhances STAT3 activity by increasing tyrosine phosphorylation of STAT3 [25]. The growth and death of gliomas are closely related to STAT3 [26, 27]. Currently, there is controversy over the role of STAT3 in pyroptosis. Some studies propose that STAT3 can activate pyroptosis, such as inducing the transition from cell apoptosis to pyroptosis through PD-L1 [28]. However, other studies suggest that STAT3 can inhibit pyroptosis, as the Src signal suppresses chemotherapy-induced typical cell pyroptosis through the STAT3 signal [29].

In our study, we explore the EZH2–STAT3 signaling axis in pyroptosis of glioma cells through in vitro experiments, validating the relationship between EZH2, STAT3, and pyroptosis in glioma cells.

## Results

### DZNep inhibits GBM cell functions and induces cell death

**Fig. 1:**
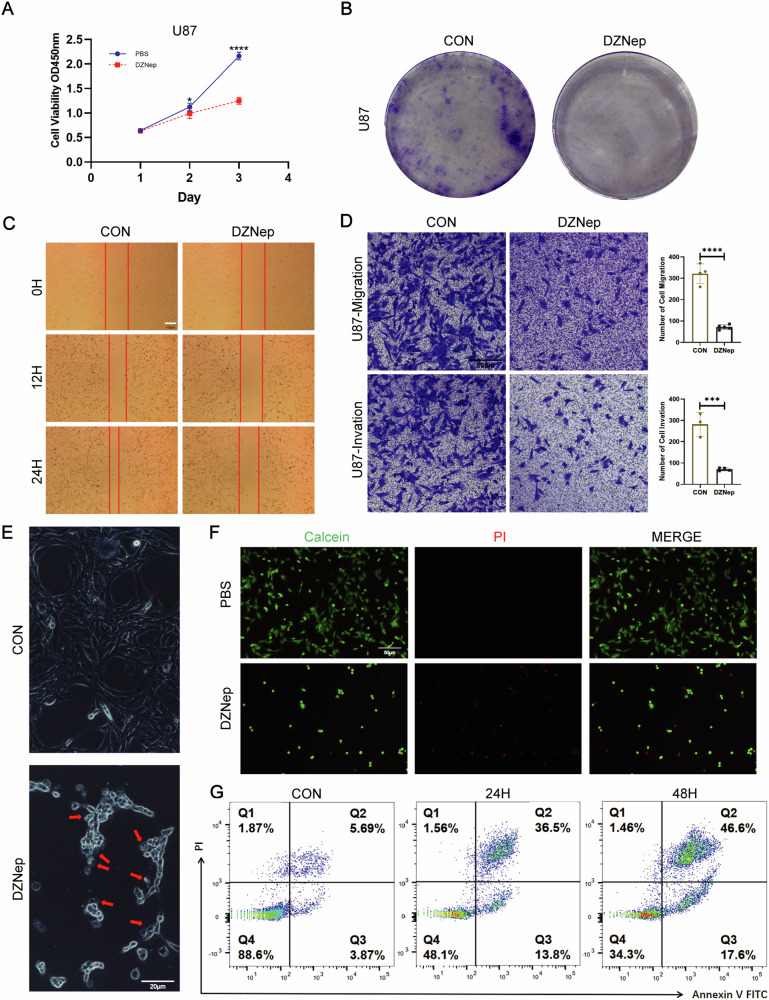
DZNep inhibits GBM cell functions and induces cell death. **A** DZNep suppresses U87 cell viability. **B** DZNep inhibits colony formation in U87 cells. **C** DZNep hinders U87 cell migration. **D** Transwell images demonstrate reduced migration and invasion of U87 cells upon DZNep treatment. **E** Apoptotic features observed under light microscopy after DZNep treatment. Red arrows indicate apoptotic bubbles. **F** PI staining reveals the count of dead and live U87 cells after DZNep treatment. **G** Cell apoptosis assays indicate the number of Annexin V–PI-positive cells during the DZNep treatment process. For **D**–**G**, data are presented as mean ± SD, *p < 0.05, ***p < 0.001, ****p < 0.0001, N.S. not significant by *t*-test.

To assess the changes in the viability of GBM cells after inhibiting EZH2, we used DZNep at a concentration of 20 μM to measure the cell viability of U87. CCK-8 assays revealed a significant reduction in U87 cell viability on the third day upon DZNep treatment (Fig. 1A). Subsequently, in the Colony Formation Assay, DZNep significantly decreased the number of colonies formed by U87 cells (Fig. 1B). In the wound healing experiment with U87, we observed a significantly wider scratch width in the DZNep-treated group compared to the control group (Fig. 1C). In the Transwell assay, the migration ability of U87 cells through gaps and Matrigel was significantly lower in the DZNep-treated group compared to the control group (Fig. 1D). Microscopic observation revealed typical pyroptotic bubble morphology in U87 cells after DZNep treatment (Fig. 1E). Subsequent Calcein/PI staining demonstrated there were more dead cells and a significant decrease in viable cells after DZNep treatment. Flow cytometry analysis further confirmed that a portion of the dead cells after DZNep treatment belonged to the apoptotic category (Fig. 1F, G). In summary, DZNep significantly inhibits GBM cell functions and induces apoptosis.

### Correlation Among EZH2, STAT3, cell cycle, and pyroptosis

**Fig. 2:**
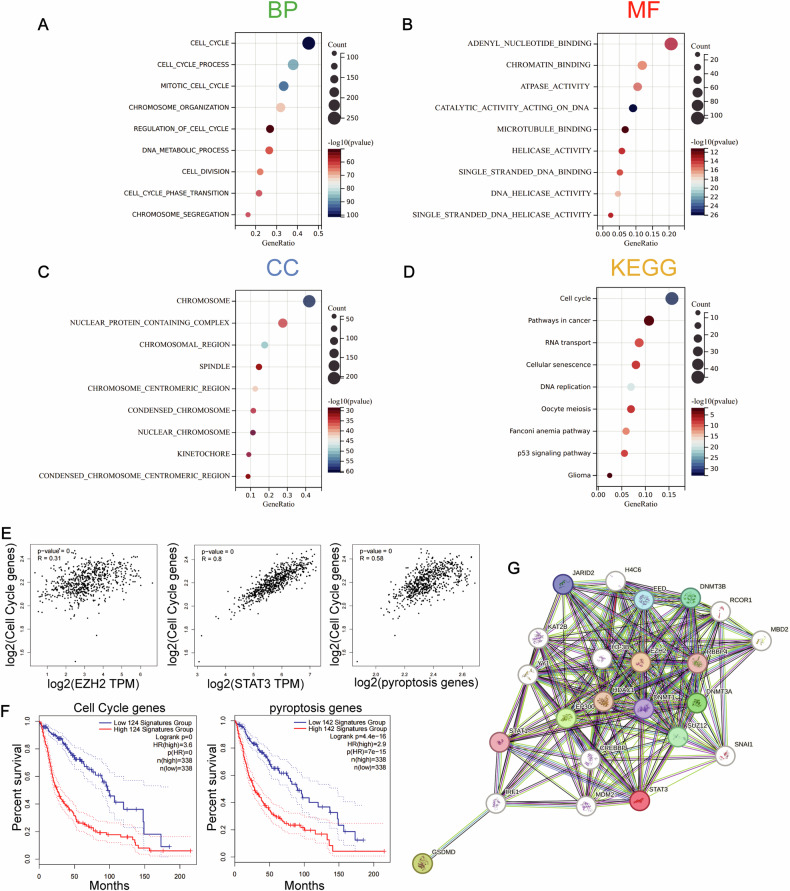
Correlation Among EZH2, STAT3, cell cycle, and pyroptosis. **A** Biological processes (BP), **B** molecular functions (MF), and **C** cellular components (CC) are mostly related to EZH2 in the TCGA database. **D** Kyoto Encyclopedia of Genes and Genomes (KEGG) pathway analysis of EZH2 in the TCGA database. **E** Correlation analysis of EZH2 and apoptosis gene set, STAT3 and apoptosis gene set, apoptosis gene set, and cell cycle gene set. **F** Kaplan–Meier curves for cell cycle and apoptosis in GBM + LGG. **G** Protein–protein interaction (PPI) network analysis for EZH2, STAT3, and GSDMD.

In the earlier stages, we observed pyroptotic bubbles under the microscope. To explore the related biological functions of EZH2, genes most correlated with EZH2 were screened using Pearson correlation analysis in the TCGA database. We conducted GO and KEGG enrichment analyses, and the cell cycle shows a strong correlation with EZH2 (Fig. 2A, D). Correlation analysis showed a connection between EZH2, STAT3, and the cell cycle gene set. Moreover, the cell cycle gene set was correlated with the pyroptosis gene set (Fig. 2E), both of which have clinical significance, high expression group patients have lower survival curves compared to the low expression group (Fig. 2F). STRING analysis indicated interactions among EZH2, STAT3, and GSDMD (Fig. 2G). These results suggest a potential connection between EZH2, STAT3, and pyroptosis that requires further investigation.

### EZH2 as an upstream regulator of STAT3, regulates pyroptosis through the inflammasome

**Fig. 3:**
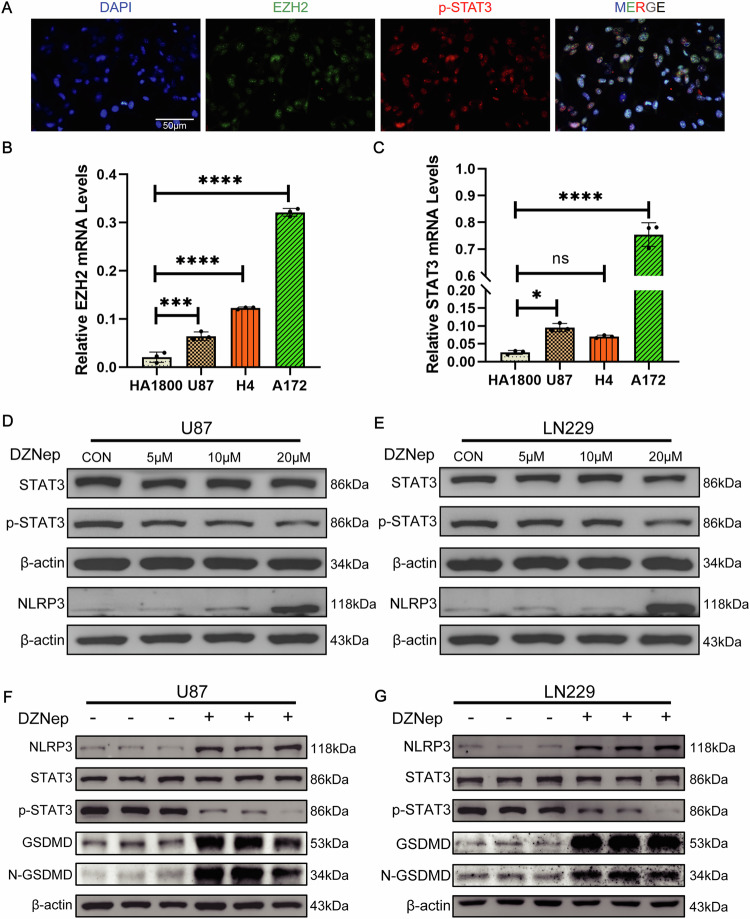
EZH2, as an upstream regulator of STAT3, regulates pyroptosis through the inflammasome. **A** Fluorescence microscopy observes the localization of EZH2 (green) and p-STAT3 (red) in U87. **B**, **C** RT-qPCR detects the expression levels of EZH2 and STAT3 in HA1800, U87, H4, and A172. **D**, **E** Protein blotting shows the protein levels of STAT3, p-STAT3, and NLRP3 in U87 and LN229 after DZNep treatment. **F**, **G** Protein blotting reveals increased protein levels of NLRP3, GSDMD, and N-GSDMD in U87 and LN229 after DZNep treatment. For **D**–**G**, data are presented as mean ± SD, *p < 0.05, **p < 0.01, ***p < 0.001, ****p < 0.0001, N.S. not significant by *t*-test.

We delved deeper into the connection between EZH2 and STAT3, particularly focusing on p-STAT. Immunofluorescence (IF) staining confirmed co-localization of EZH2 (green) and p-STAT3 (red) in U87, mainly concentrated in the cell nucleus (Fig. 3A). Compared to the normal glioma cell line HA1800, quantitative PCR (qPCR) analysis revealed EZH2 and STAT3 were higher in U87, H4, and A172 (glioma cell lines) (Fig. 3B, C). Furthermore, after DZNep treatment, the total STAT3 in U87 and LN229 did not show significant changes, while p-STAT3 decreased as the dosage increased (Fig. 3D). Simultaneously, the inflammasome component NLRP3 showed increased protein levels after DZNep treatment, with a significant elevation at 20 μM (Fig. 3D). Similarly, pyroptosis proteins GSDMD and N-GSDMD exhibited significantly increased protein levels after DZNep treatment (20 μM) (Fig. 3E, F). These findings suggest a reciprocal relationship between EZH2 and STAT3, allowing them to regulate glioma pyroptosis.

### Inhibition of STAT3 activates pyroptosis and suppresses glioma cell proliferation

**Fig. 4:**
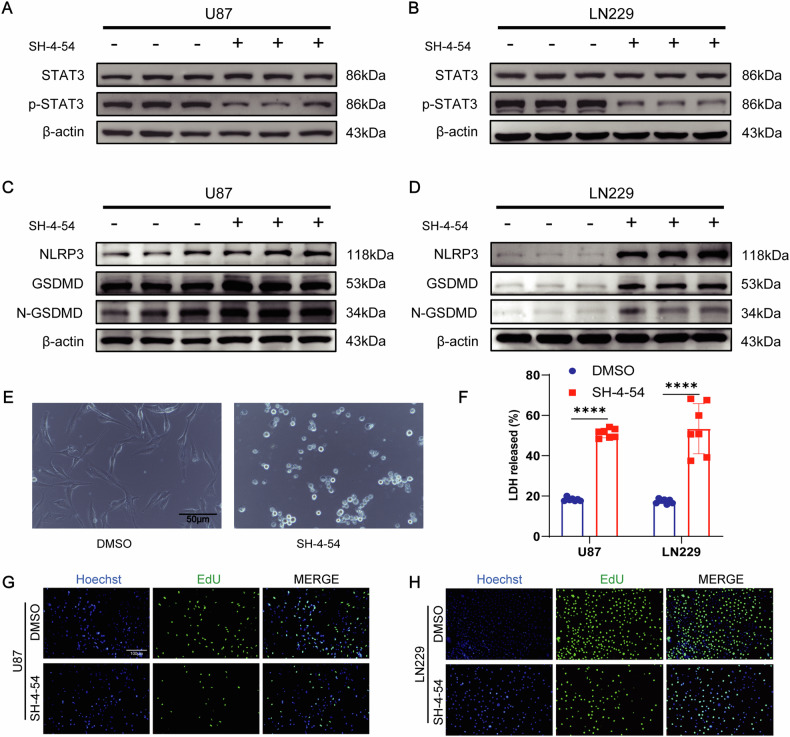
Inhibition of STAT3 activates pyroptosis and suppresses glioma cell proliferation. **A**, **B** Detection of STAT3 and p-STAT3 expression levels in cells after 48 h treatment with SH-4–54 (10 μM). **C**, **D** Protein blotting shows the protein levels of NLRP3, GSDMD, and N-GSDMD in U87 and LN229 after SH-4–54 treatment. **E** Morphological changes in U87 cells after SH-4–54 treatment. **F** Increased lactate dehydrogenase (LDH) levels in the culture medium after 48 h SH-4–54 treatment. **G**, **H** EdU assay demonstrates decreased proliferation of U87 and LN229 cells after SH-4–54 treatment. For **A**–**D**, data are presented as mean ± SD, *p < 0.05, **p < 0.01, ***p < 0.001, ****p < 0.0001, N.S. not significant by *t*-test.

According to previous research, EZH2 can regulate the phosphorylation level of STAT3 and activate pyroptosis in glioma cells through the inflammasome. Subsequently, we intervened using the STAT3 inhibitor SH-4–54, and SH-4–54 (10 μM) could inhibit the expression of p-STAT3 protein (Fig. 4A, B). It also had the ability to increase the protein expression levels of activated NLRP3, GSDMD, and N-GSDMD (Fig. 4C, D). Under an optical microscope, we observed significant pyroptotic bubbles in U87 cells after SH-4–54 treatment (Fig. 4E). The lactate dehydrogenase (LDH) content in the culture medium of U87 cells was significantly increased in the SH-4–54 group (Fig. 4F). Using EdU to detect the proliferation of glioma cell lines, SH-4–54 could reduce the proliferation of U87 and LN229 (Fig. 4G, H).

### EZH2–STAT3 activation of pyroptosis accompanied by inflammatory factor production

**Fig. 5:**
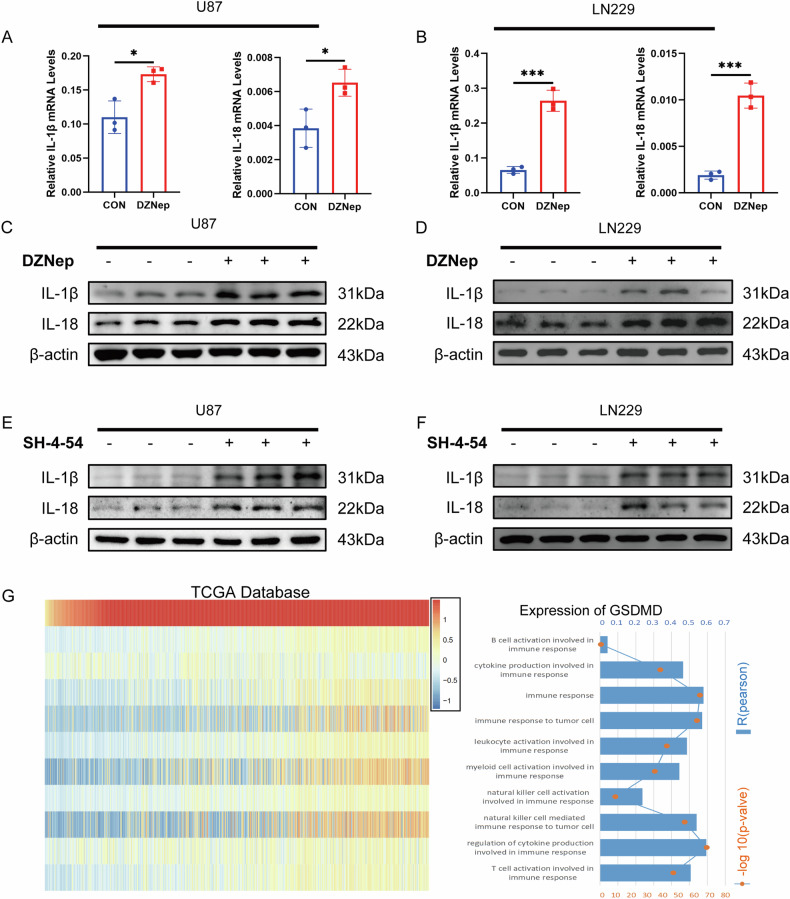
EZH2–STAT3 activation of pyroptosis accompanied by inflammatory factor production. **A**, **B** RT-qPCR detects IL-1β and IL-18 expression levels in U87 and LN229 cells after DZNep treatment. **C**–**F** Protein blotting shows IL-1β and IL-18 protein levels in U87 and LN229 cells after DZNep and SH-4–54 treatment. **G** Heatmap displaying GSDMD expression and immune function enrichment scores in TCGA databases. *R*‐value and *P*‐value of the correlation analysis are shown on the right. For **C**–**F**, data are presented as mean ± SD, *p < 0.05, **p < 0.01, ***p < 0.001, N.S. not significant by t-test.

Pyroptosis is a form of cell death, often associated with inflammatory factors, mainly IL-1β and IL-18. After treating U87 and LN229 cells with DZNep, the RNA expression levels of IL-1β and IL-18 were significantly increased. Similarly, the protein content of IL-1β and IL-18 also showed similar results (Fig. 5A–D). Subsequently, after inhibiting STAT3 with SH-4-54, IL-1β, and IL-18 protein expression levels also saw significant increases (Fig. 5E, F). Inflammation often accompanies immune responses, and to further explore the association between pyroptosis and immune cell infiltration, GSVA analysis showed that with the increase of GSDMD, glioma tissues often had more immune cell infiltration and inflammatory responses (Fig. 5G).

### RO8191 Reverses SH-4–54-mediated glioma cell death and pyroptosis

**Fig. 6:**
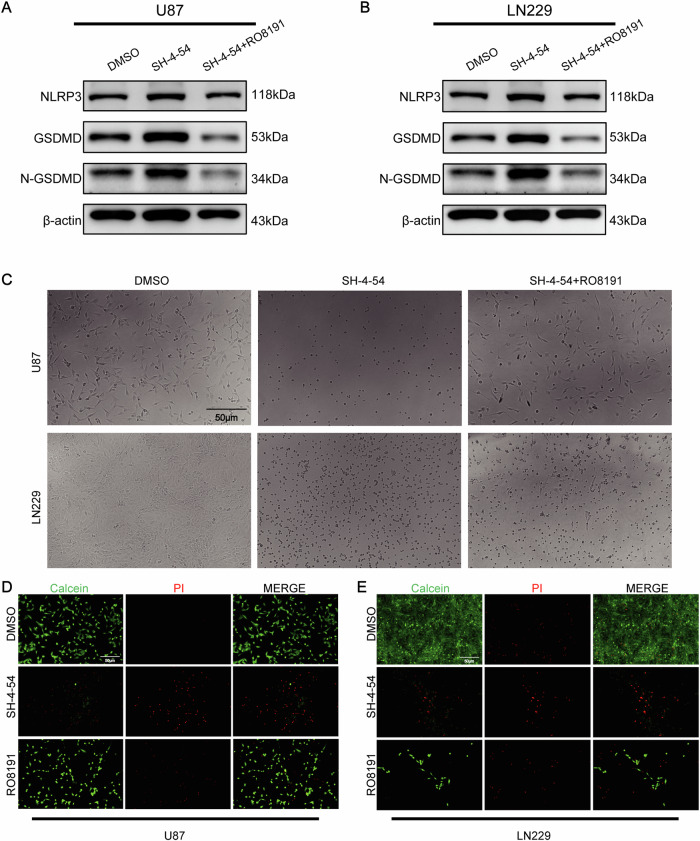
RO8191 reverses SH-4–54-mediated glioma cell death and pyroptosis. **A**, **B** Immunoblotting shows protein levels of NLRP3, GSDMD, N-GSDMD in control, SH-4–54, and SH-4–54 + RO8191 groups after SH-4–54 treatment. **C** Microscopic observation of the morphological effects of RO8191 on U87 and LN229 cells after SH-4–54 treatment. **D**, **E** EdU assay demonstrates proliferation capabilities in control, SH-4–54, and SH-4–54 + RO8191 groups. For **A**, **B**, data are presented as mean ± SD, *p < 0.05, **p < 0.01, ***p < 0.001, ****p < 0.0001, N.S. not significant by ANOVA.

Previous studies demonstrated that STAT3, as a downstream target of EZH2, can regulate pyroptosis. Using the STAT3 agonist (RO8191, 10 μM) could reverse SH-4–54-activated pyroptosis in U87 and LN229 (Fig. 6A, B). Meanwhile, under a microscope, it was observed that SH-4–54 caused U87 and LN229 to lose their original cell morphology, while RO8191 could reverse these morphological changes, restoring the cells to their original form (Fig. 6C). Furthermore, RO8191 could reduce cell death caused by SH-4–54, with a significant decrease in dead cells in the RO8191 group compared to the SH-4–54 group (Fig. 6D, E). These results indicate that EZH2 regulates glioma cell pyroptosis and function through STAT3, and RO8191 can reverse this process.

## Discussion

Pyroptosis, as an emerging form of cell death, not only manifests distinct morphological changes with cells swelling and forming large bubbles but also typically relies on the involvement of the GSDMD protein and the inflammasome in the classical pathway [30]. Apart from the classical pathway, the non-classical pathway of pyroptosis relies on the involvement of caspase-4/5/11 [31, 32]. However, the signaling pathways governing tumor pyroptosis currently lack a theoretical basis. Research on tumor pyroptosis is predominantly centered around cancer drugs, such as anthocyanin and docosahexaenoic acid (DHA) [33, 34], both of which can induce tumor pyroptosis through the classical pathway. As research progresses, mounting evidence suggests a close correlation between tumor pyroptosis and tumor immunity. In tumors exhibiting immune cell infiltration, a high expression of GSDMD and GSDME often accompanies this phenomenon, indicating tumor pyroptosis and the immune response exhibit a mutually reinforcing positive feedback mechanism. Despite the relatively limited research on pyroptosis in gliomas, bioinformatic analyses have affirmed pyroptosis as a crucial indicator of immune microenvironment infiltration in gliomas, offering a potential direction for subsequent glioma treatments [35, 36].

Prior investigations have established that EZH2 can stimulate glioma growth. EZH2 exerts its influence on gliomas through methylation and involvement in multiple signaling pathways that regulate glioma development. Studies propose that EZH2 can enhance glioma resistance to temozolomide (TMZ) by regulating the FADD/PARP1 axis [37]. Additionally, there is evidence of a reciprocal binding relationship between EZH2 and STAT3, where EZH2 modulates the activity of p-STAT3 through STAT3 methylation [38]. Our findings indicate that EZH2, serving as an upstream regulator of STAT3, can modify STAT3 activity by influencing EZH2 levels. While there is currently a dearth of research on the regulation of glioma pyroptosis by EZH2, our initial experiments demonstrate that treatment with the EZH2 inhibitor DZNep substantially activates the expression of GSDMD in glioma cells, concomitant with an increase in inflammatory factor expression. These findings imply that EZH2 might play a crucial role in initiating glioma cell pyroptosis.

In recent years, there has been a surge in research on STAT3 and pyroptosis, suggesting that STAT3 may constitute a component of the signaling pathway regulating glioma pyroptosis. Recent studies also indicate that inhibiting STAT3 can activate cell pyroptosis and elicit anti-tumor immune responses [39]. These findings propose pyroptosis as a potential regulatory target of STAT3, with inhibiting STAT3 potentially activating glioma pyroptosis. Our prior studies have affirmed the close association between EZH2 and the cell cycle, as well as the correlation between the cell cycle and pyroptosis. Subsequent experiments have demonstrated that inhibiting EZH2 reduces the protein content of p-STAT3, significantly increases pyroptosis-related proteins GSDMD and NLRP3, and reveals pyroptotic bubbles under a microscope. Further experiments have substantiated the regulation of glioma pyroptosis by EZH2 through STAT3 inhibition (SH-4–54) and recovery (RO8191). Moreover, we have illustrated that the EZH2–STAT3 signaling pathway governs glioma proliferation, migration, invasion, apoptosis, and pyroptosis. These findings present a potential therapeutic strategy for future glioma patient treatments.

## Conclusion

**Fig. 7:**
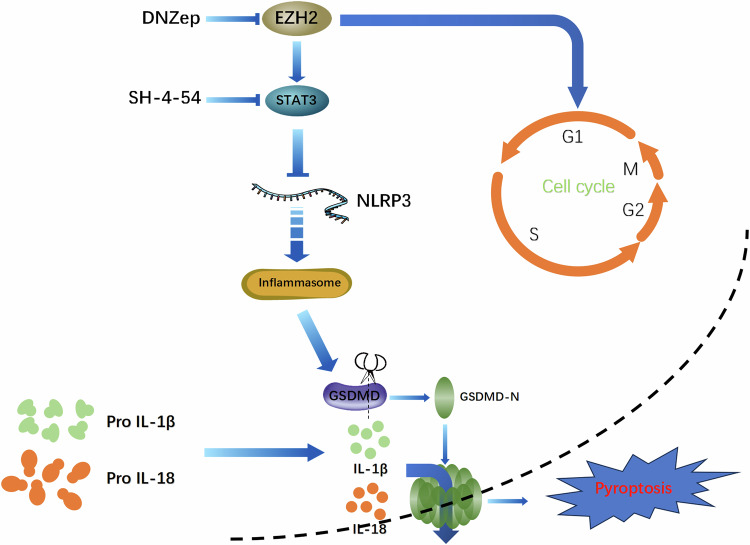
Schematic diagram of DZNep regulates pyroptosis in glioblastoma through the EZH2–STAT3 signaling pathway. In glioma cells, EZH2-related genes are closely associated with the cell cycle. EZH2 regulates the expression of the inflammasome NLRP3 via the STAT3 signaling pathway. By inhibiting STAT3, NLRP3 can be activated to cleave GSDMD into its N-GSDMD form. The cleaved GSDMD then translocates to the cell membrane to form pores, leading to apoptosis in glioma cells. This process is accompanied by the production of significant amounts of IL-1β and IL-18.

In this study, EZH2 was introduced for the first time into the realm of glioma pyroptosis. The use of the EZH2 inhibitor DZNep not only inhibits the occurrence and development of glioma but also activates glioma pyroptosis. Simultaneously, we demonstrated that inhibiting p-STAT3 through EZH2 enhances NLRP3 inflammasome activity, promoting glioma pyroptosis and the expression of inflammatory factors IL-1β and IL-18 (Fig. 7). Our findings provide a potential signaling pathway for the study of glioma pyroptosis.

## Materials and methods

### Cell culture and treatment

Human glioblastoma cell lines HA1800, U87, LN229, A172, and H4 were obtained from ProCell Life Science. (Wuhan, China). U87 and LN229 cells were cultured in Dulbecco’s Modified Eagle Medium (DMEM) (10% fetal bovine serum (FBS) + 1% penicillin–streptomycin (P/S)). All cells were cultured in a 5% CO_2_ incubator at 37 °C.

### Antibodies and reagents

Antibodies were sourced from the following providers: Cell Signaling Technology (Danvers, Massachusetts, USA)—anti-EZH2 (1:1000), anti-STAT3 (1:1000), anti-phospho (p)-STAT3 (1:1000); Affinity Biologicals (Jiangsu, China)—anti-NLRP3 (1:1000); Abcam (Cambridge, MA, USA)—anti-GSDMD (1:1000), anti-cleaved N-terminal GSDMD (1:1000); Proteintech (Wuhan, China)—Beta Actin Recombinant antibody (1:20,000), IL-1 Beta Polyclonal antibody, IL-18 Polyclonal antibody; secondary antibodies (goat anti-rabbit and goat anti-mouse). Inhibitors and agonists were purchased from MedChemExpress (New Jersey, USA) - DZNep and RO8191, while SH-4–54 was purchased from Selleck (Houston, Texas, USA). CCK-8, DMSO, and Goat Serum were from Biosharp (Anhui, China). The polyvinylidene fluoride (PVDF) membrane and chemiluminescence reagents were provided by Millipore (Massachusetts, USA).

### Cell viability assay

To determine the effect of DZNep on U87 cell viability, the CCK-8 assay was utilized. The specific methodology adhered to previously published articles by our research group.

### Colony formation assay

U87 cells were seeded at a density of 500 cells per well in a 6-well plate. After the cells were stabilized, they were treated with DZNep (20 μM) and DMSO control. The cells were cultured for 48 h, followed by replacement with fresh medium and continued growth for one week. Subsequently, the samples underwent blocking and staining, and photographs were taken for documentation.

### Wound healing assay

U87 cells were seeded in six-well plates until reaching approximately 80% confluence. Horizontal cross-shaped scratches were made in each well using a 200 μl pipette tip. After washing away detached cells, a fresh medium containing DZNep (20 μM) and DMSO control was added. Images were captured at 0, 12, and 24 h to observe changes in scratch width.

### Calcein-AM/propidium iodide (PI) staining

Calcein-AM/PI staining was performed using the Cell Viability/Cytotoxicity Assay Kit (Beyotime). The specific procedure followed the manufacturer’s instructions or referenced previously published articles by our research group. A fluorescence microscope (Zeiss, Germany) was utilized to capture the images.

### Flow cytometry

U87 cells were cultured to the appropriate density and treated with DZNep (20 μM) and DMSO separately for 48 h. Cells were then collected, and an Apoptosis Detection Kit (Beyotime) was employed for apoptosis detection. The procedure followed the manufacturer’s instructions, and flow cytometry analysis was conducted with a BD Biosciences flow cytometer. Data analysis was conducted using FlowJo.

### Transwell assays

Transwell assays were conducted using 8 mm polycarbonate membrane inserts (Corning) coated with Matrigel matrix (Corning) for invasion assays. Please refer to the specific protocol outlined in the relevant articles from our research group, and the results were recorded using an inverted microscope.

### Immunofluorescent staining

After seeding U87 cells, fixation and permeabilization were carried out according to the instructions, followed by blocking. Specific primary antibodies targeting p-STAT3 (1:300) and EZH2 (1:300) were applied. Subsequently, corresponding fluorescent secondary antibodies were used for processing. DAPI nuclear staining with a quenching agent was performed for 5 min, followed by image capture.

### Western blotting analysis

Collect cells from both the treatment and control groups, and extract cellular proteins. Separate the proteins using appropriate SDS-PAGE gels and transfer them onto PVDF membranes. Proceed with blocking, primary antibody incubation, secondary antibody incubation, and finally, detection using enhanced chemiluminescence (ECL). Protein bands can be analyzed using ImageJ software.

### RT-qPCR

RNA was extracted using the Tissue RNA Purification Kit from Yeesen Biotech (Shanghai, China), followed by reverse transcription to generate cDNA. Quantitative PCR (qPCR) was performed using the qPCR SYBR Green Master Mix kit from Yeesen Biotech. Primers were designed using Primer3 (https://primer3.ut.ee/).

### LDH release assay

The experiment was conducted using U87 cells. Cells were treated with SH-4–54 (10 μM) and DMSO control in culture plates for 48 h. Subsequently, procedures were carried out according to the manufacturer’s instructions, with LDH release reagent and LDH detection working solution prepared freshly. During the process, certain procedures required light protection, and absorbance was measured at 490 nm.

### EdU-488 cell proliferation detection

U87 cells were cultured in a 96-well plate at an appropriate density, with control and treatment groups established. The Beyotime EdU detection kit was employed according to the manufacturer’s instructions. After fixation and permeabilization steps, click reaction solution was added, followed by staining with Hoechst 33342.

### Data and sources

All bioinformatics analysis data utilized in this study were sourced from TCGA (https://portal.gdc.cancer.gov/), primarily using glioblastoma multiforme (GBM) and low-grade glioma (LGG) datasets. Correlation analysis and Kaplan–Meier curve plotting were performed using GEPIA2 (http://gepia2.cancer-pku.cn/#index). Protein–protein interaction (PPI) network and co-expression analysis for EZH2/STAT3/GSDMD were conducted using STRING (https://string-db.org/).

### Functional enrichment analysis

To identify the genes most closely linked to EZH2 or a distinctive gene list characteristic of the cell cluster, we submitted them to the Database for DAVID. Using standard gene names, within the same species, we conducted GO and KEGG analysis. The top nine findings are displayed in ascending order based on their *P*-values (*P* < 0.05).

### Gene set enrichment analysis (GSVA)

Collecting the necessary immunological dataset for this study from AmiGO. Utilizing the provided package in the R environment under default parameters, the enrichment scores were obtained using R programming language, and the enrichment results were visually represented using heatmaps generated by the pheatmap package in the R environment. Subsequently, the correlation between GSDMD and immune processes was analyzed.

### Statistical analysis

The entire dataset in this study was obtained from three groups of samples, with each group undergoing three repeated experiments. Statistical analysis involved *t*-tests for comparisons between two groups and analysis of variance (ANOVA) for comparisons among multiple groups, followed by Tukey’s post hoc test. Differences were considered significant when the *p*-value was less than 0.05. (Statistical software: GraphPad Prism 8.0).

### Supplementary information

Western blot raw bands

Data statistics

immune gene set

## Data Availability

All original data from this study has been submitted; there are no outstanding datasets awaiting submission.
