# Supplementary figures and images for: EZH2–STAT3 signaling pathway regulates GSDMD-mediated pyroptosis in glioblastoma
Source: Cell Death Discov. 2024 Jul 28;10:341. doi: 10.1038/s41420-024-02105-0 (PMC11284224; doi:10.1038/s41420-024-02105-0)

fig3

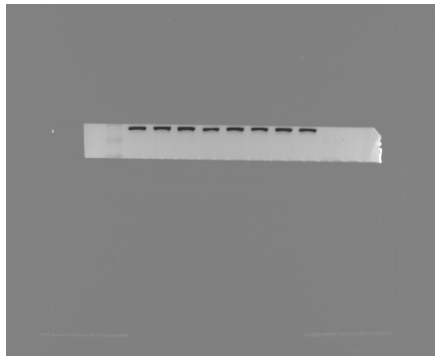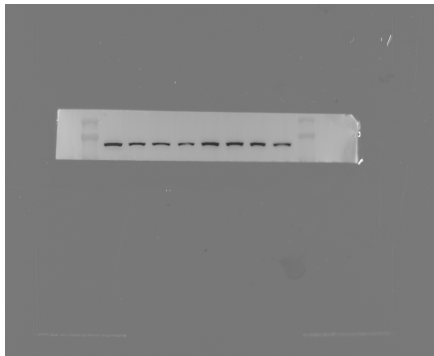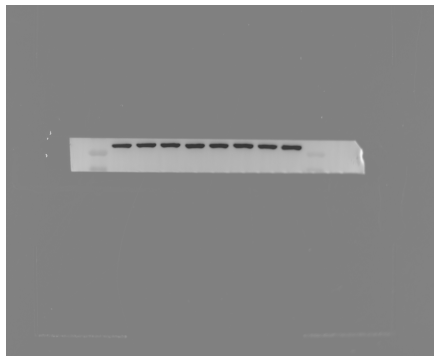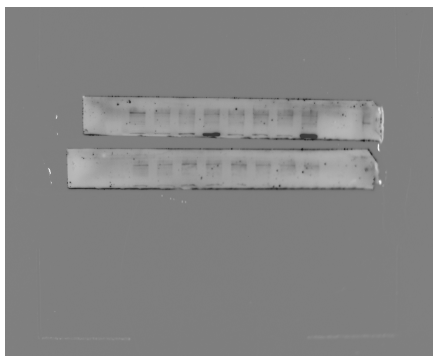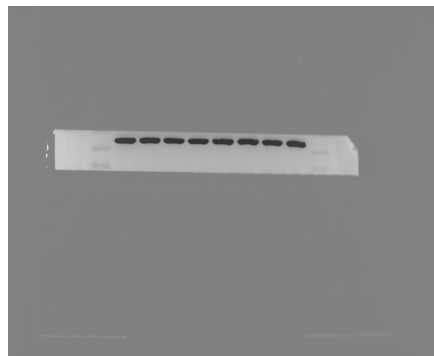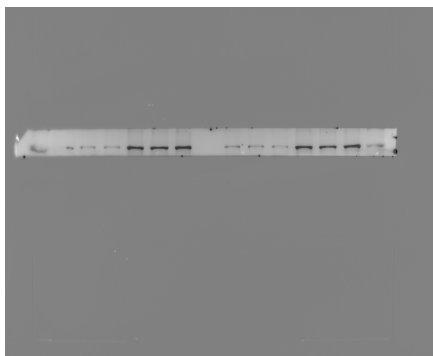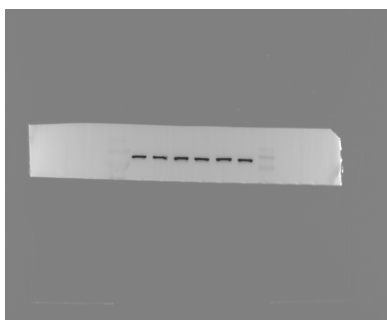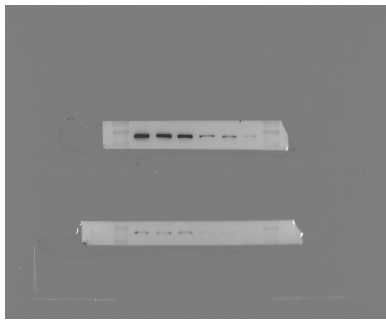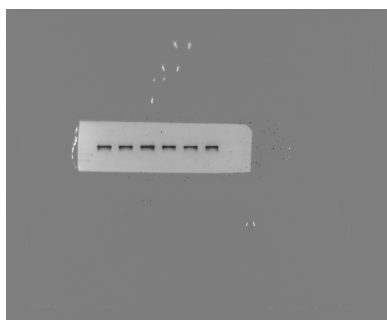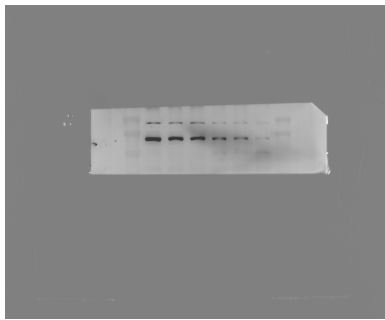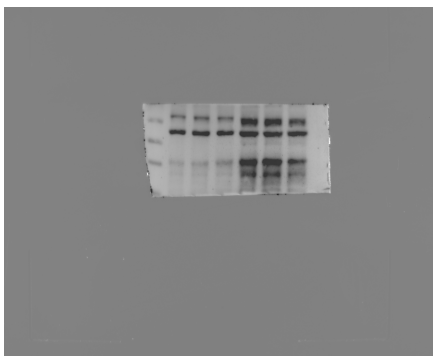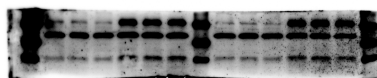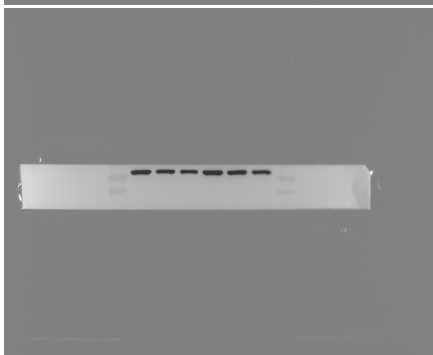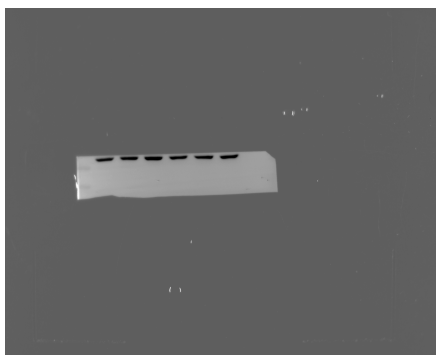

fig4

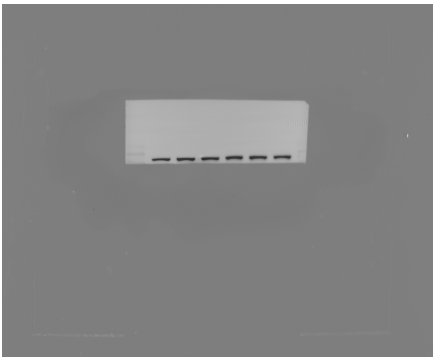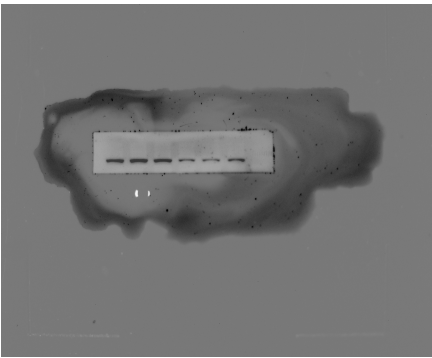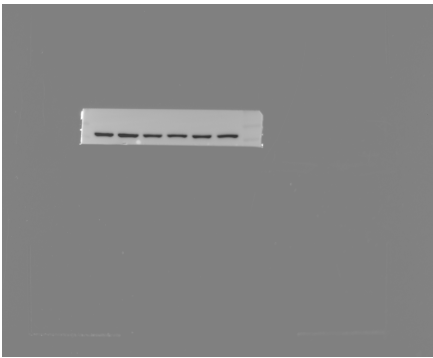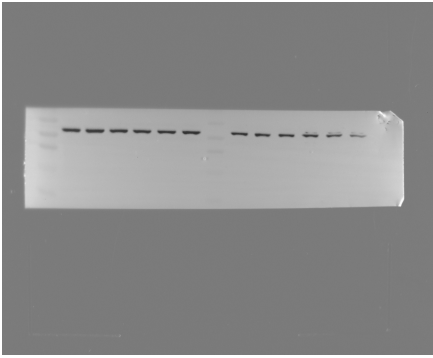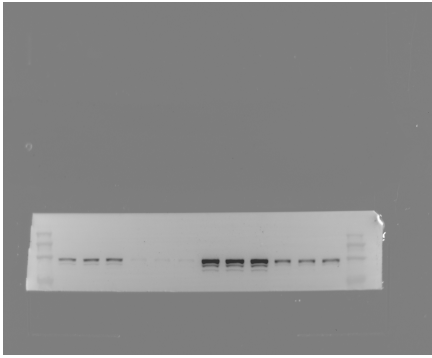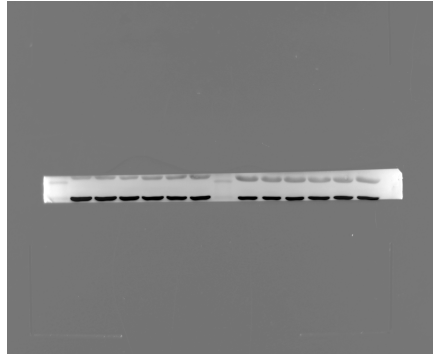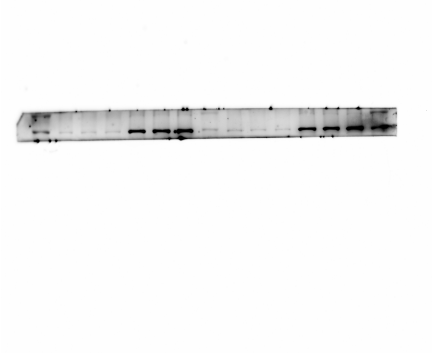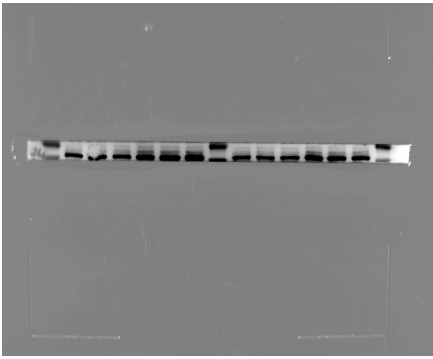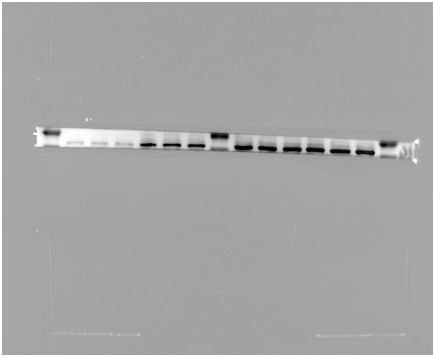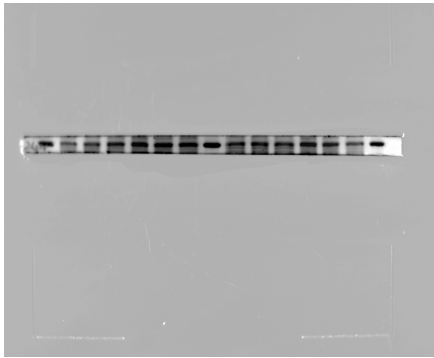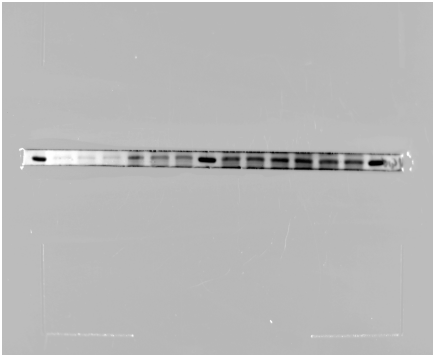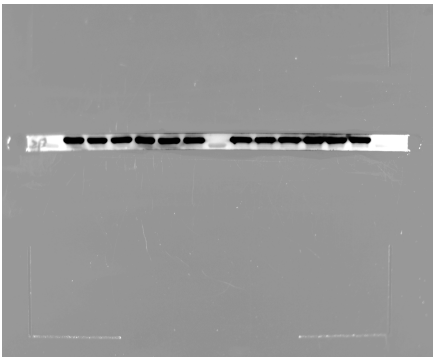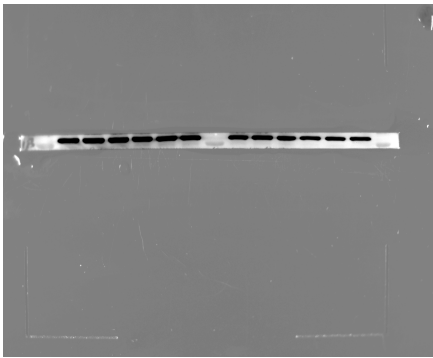

fig5

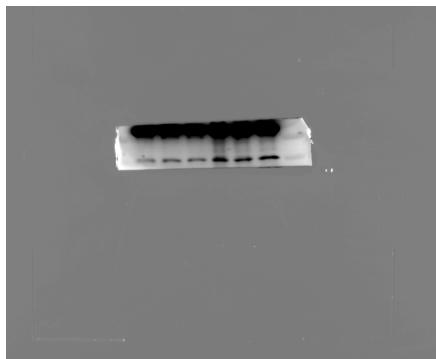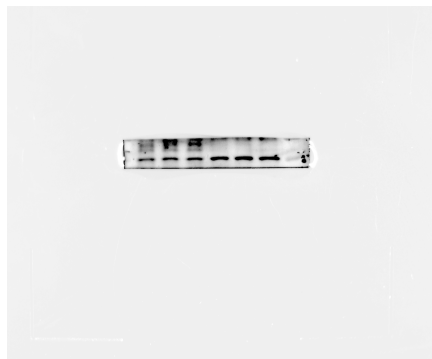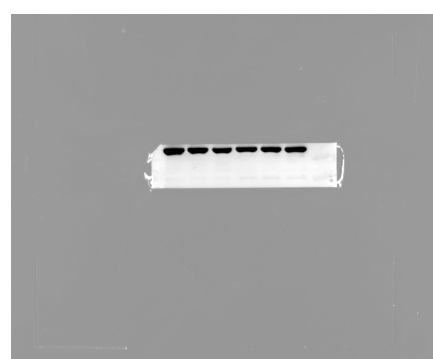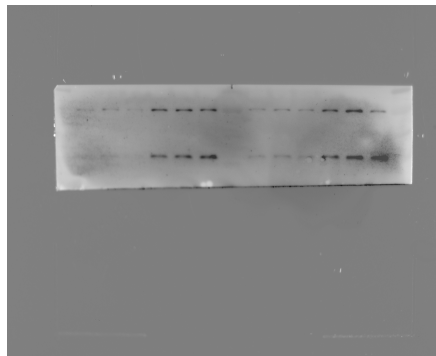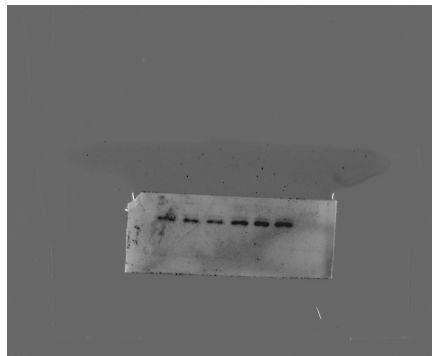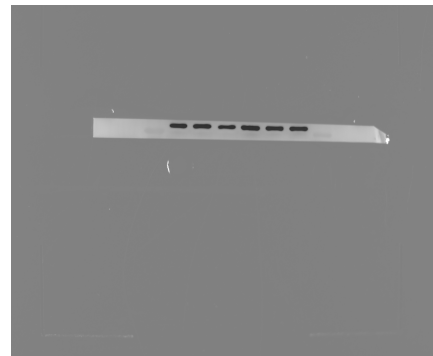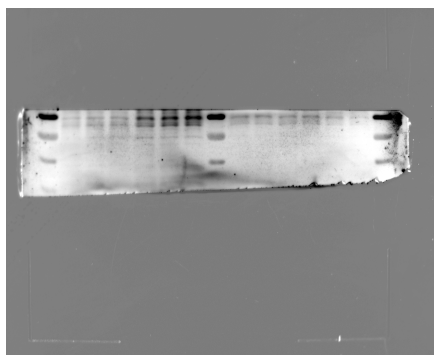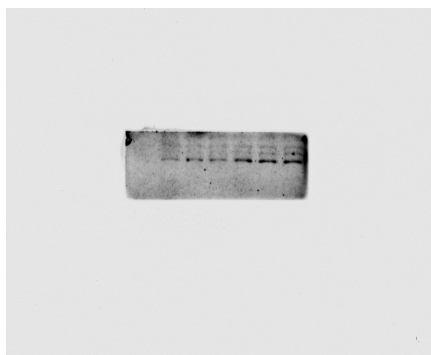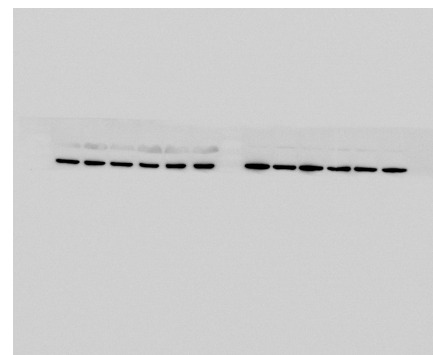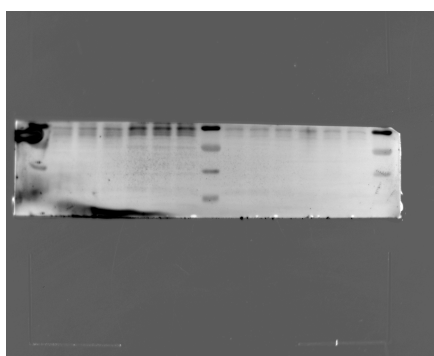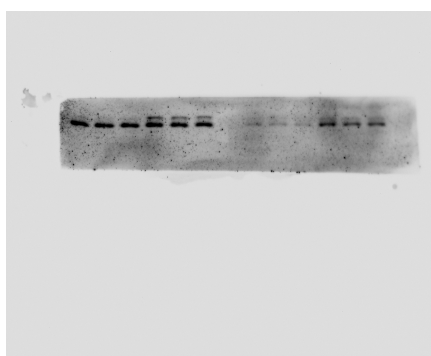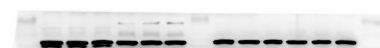

fig6

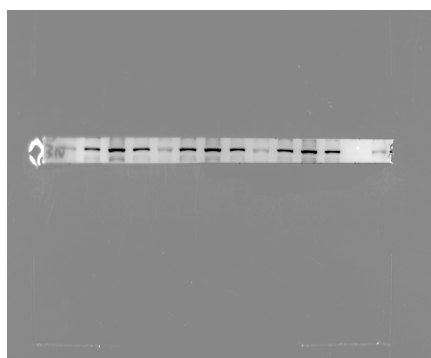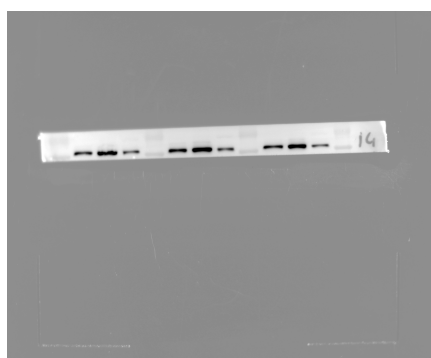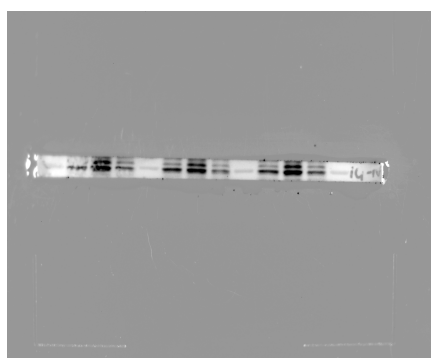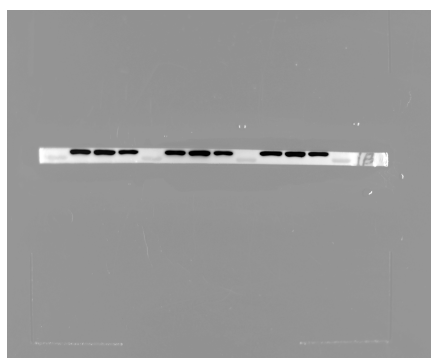

fig3

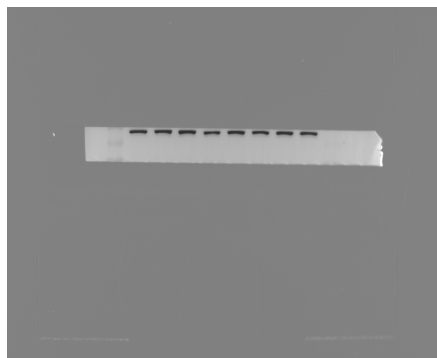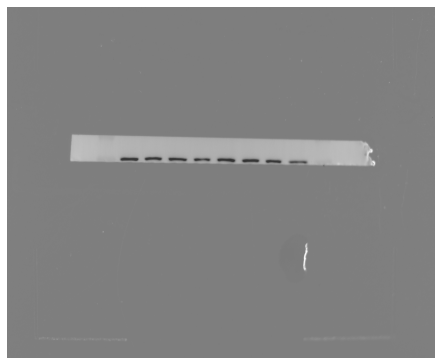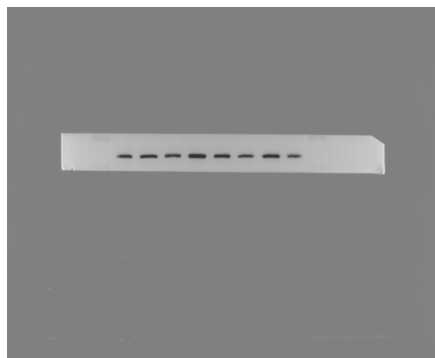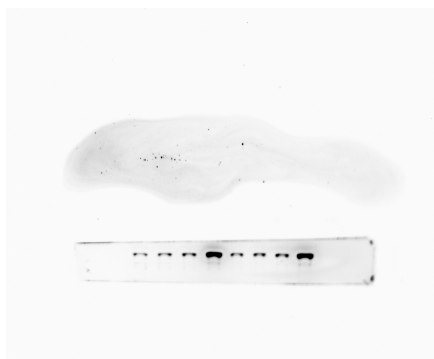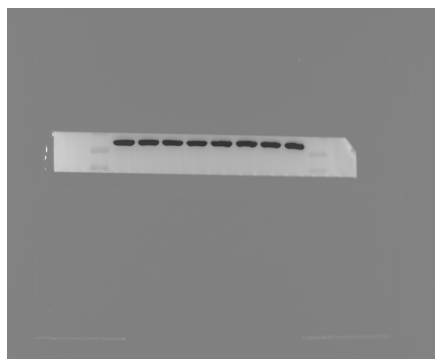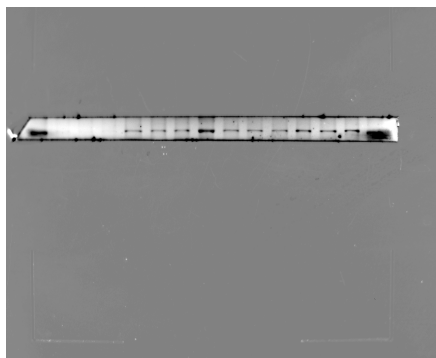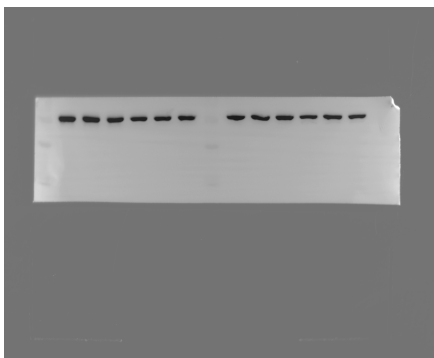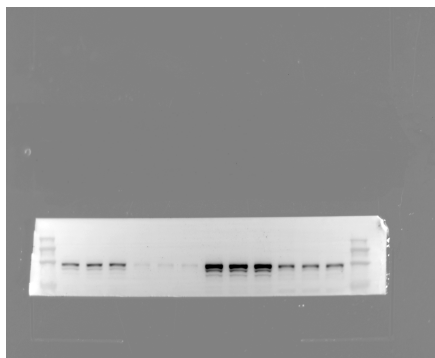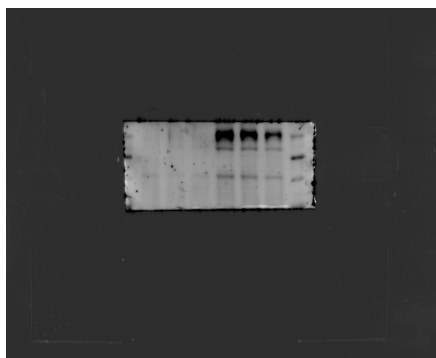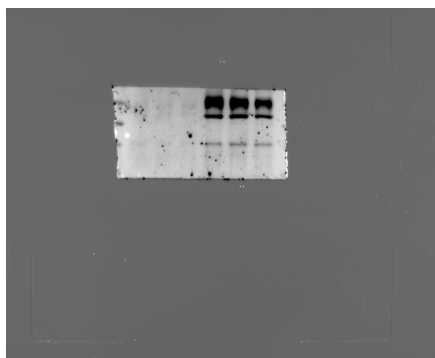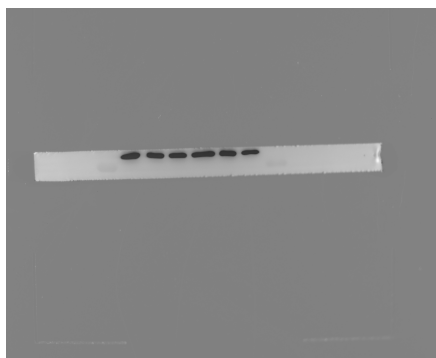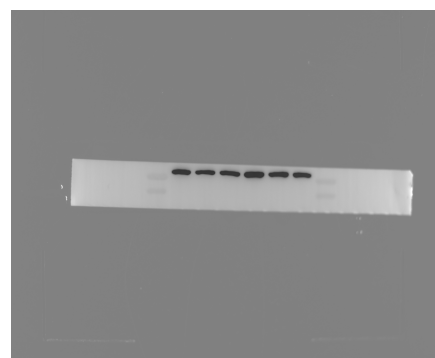

fig3

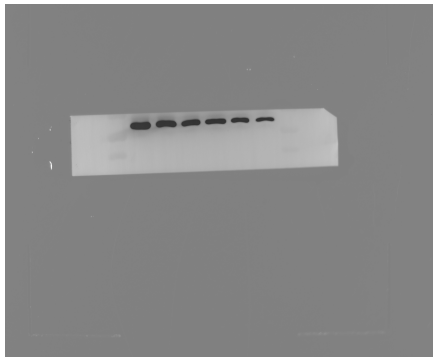

fig4

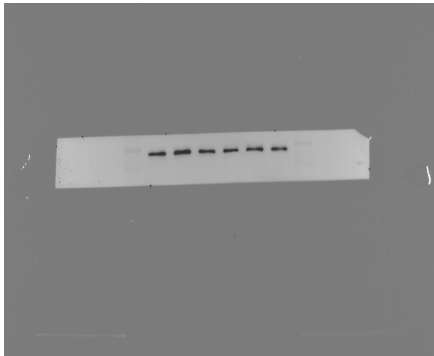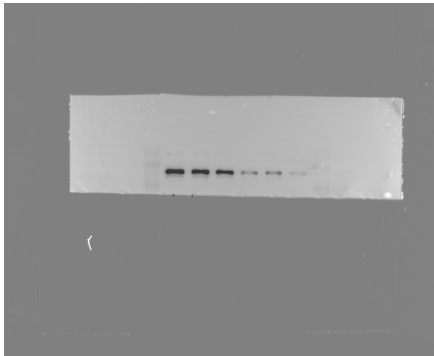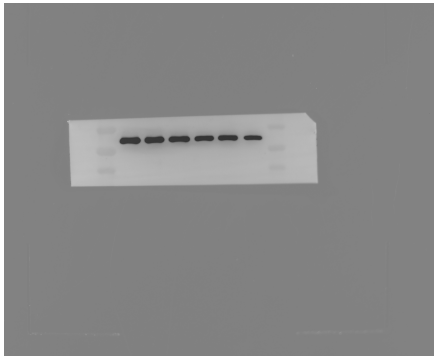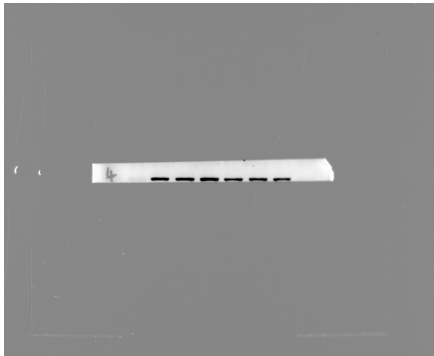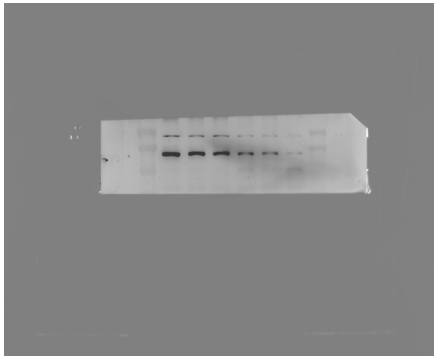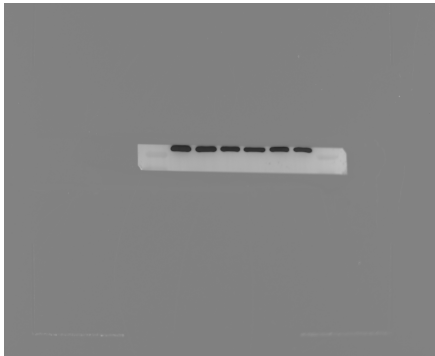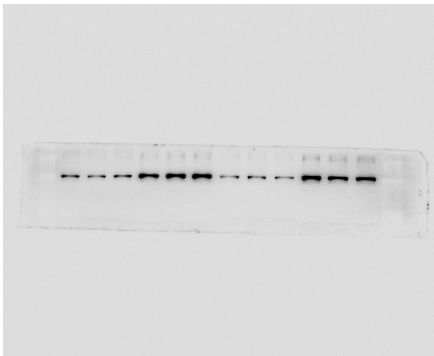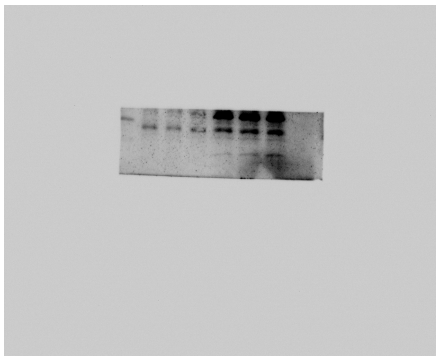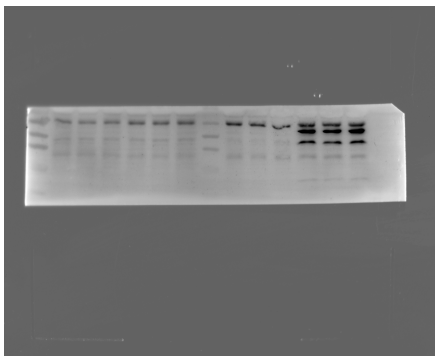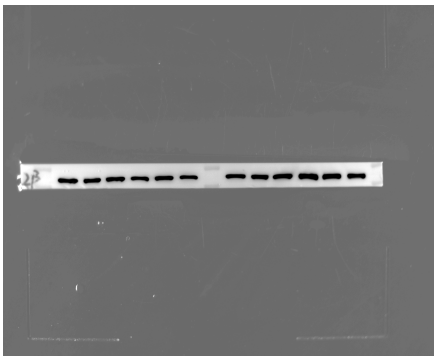

fig4

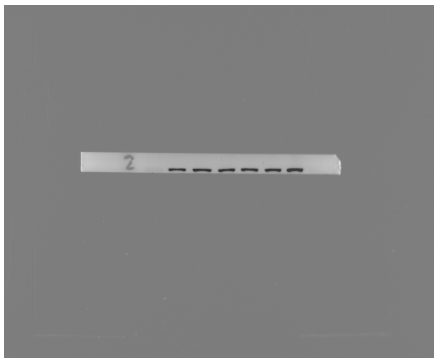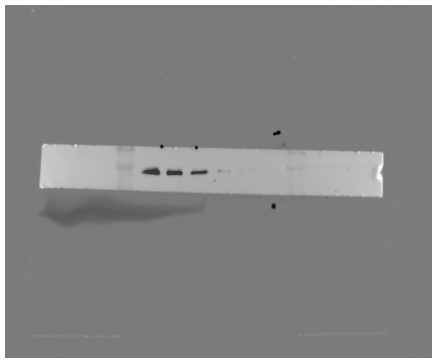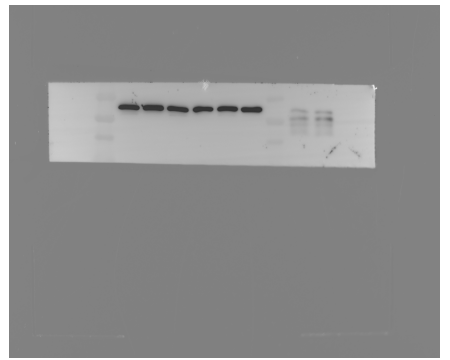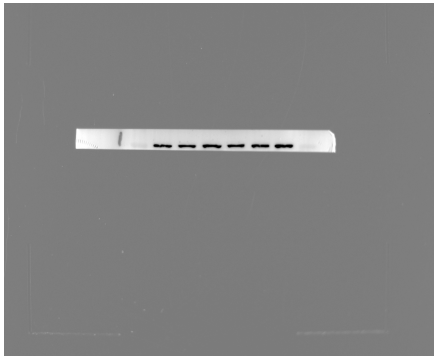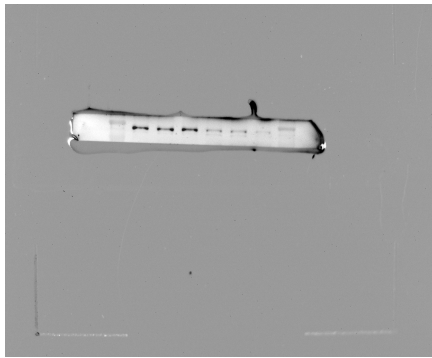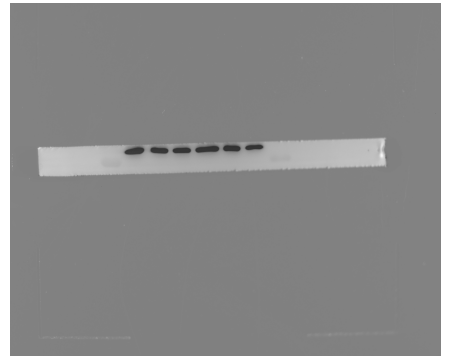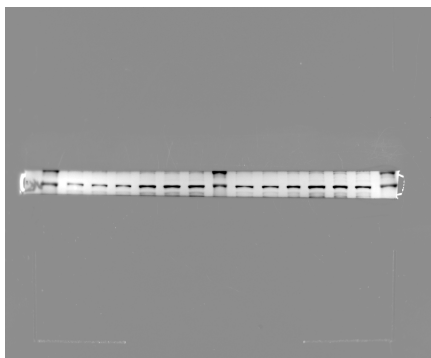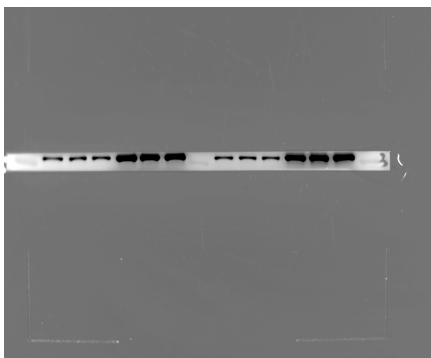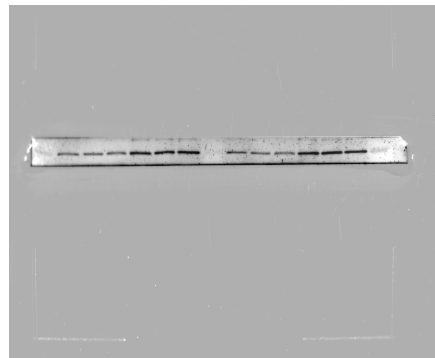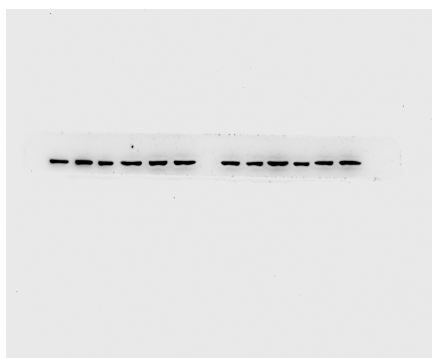

fig5

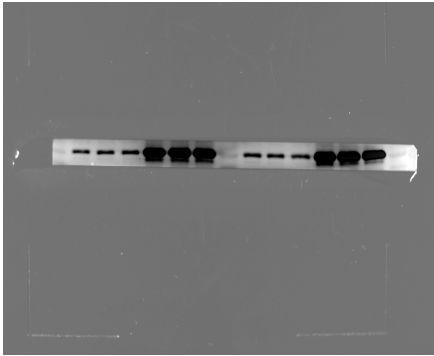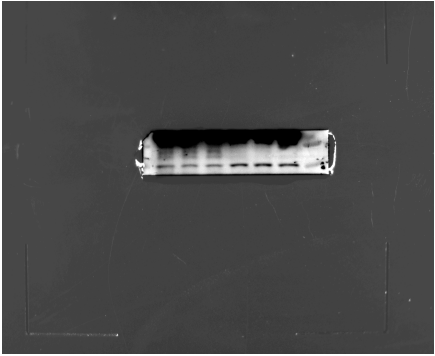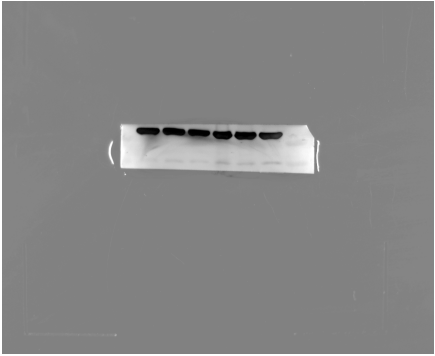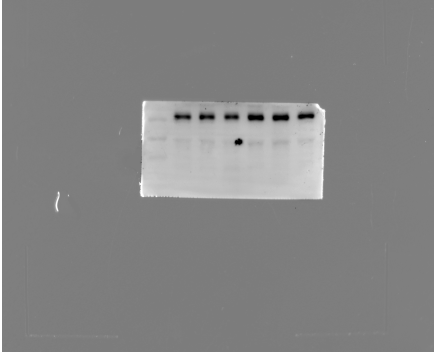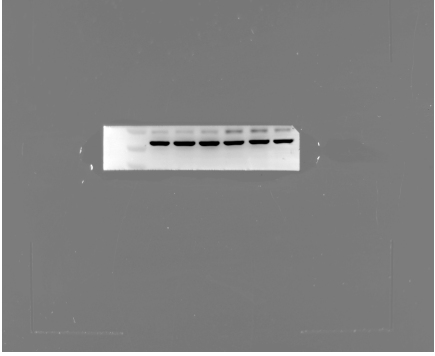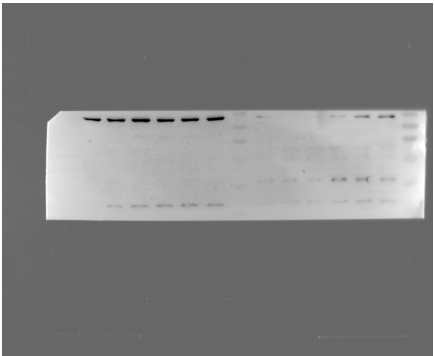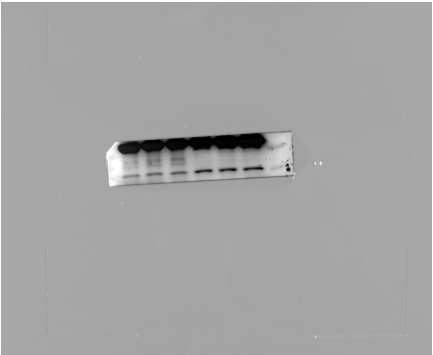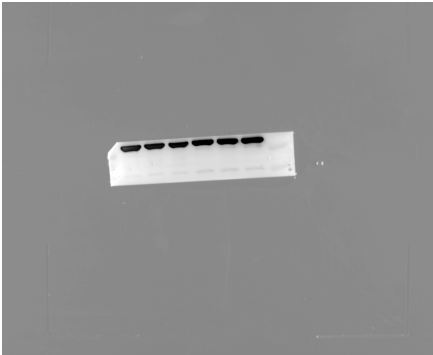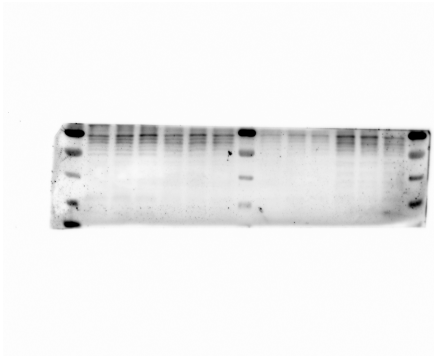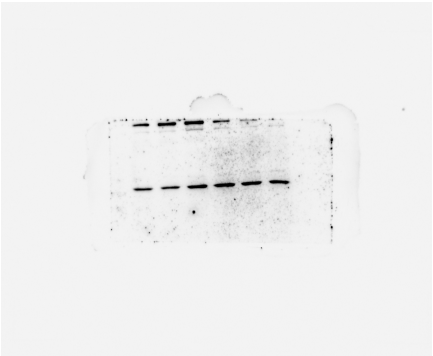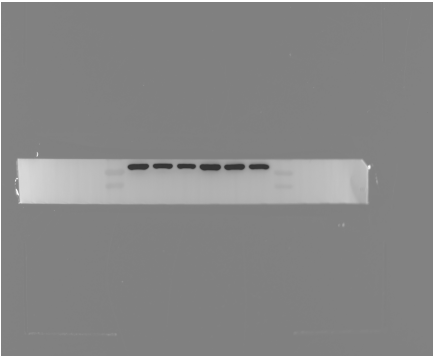

fig5

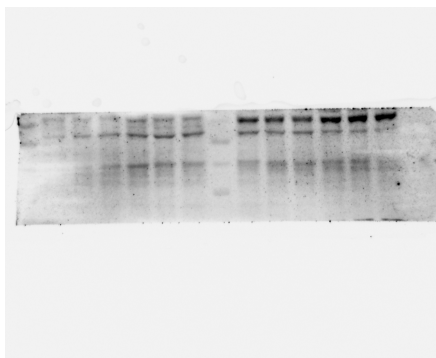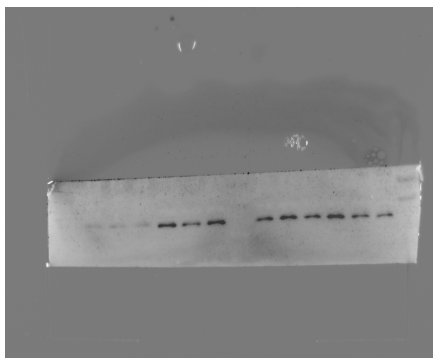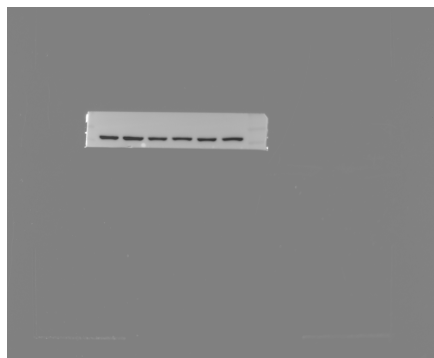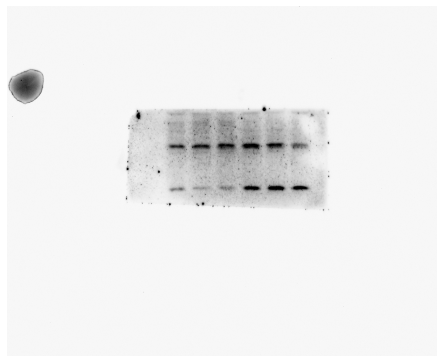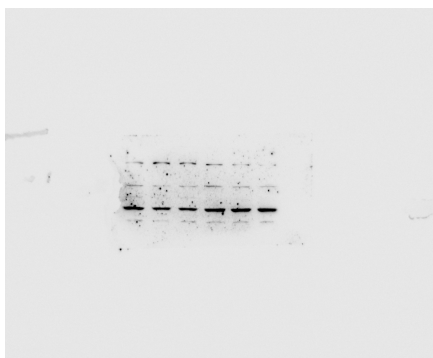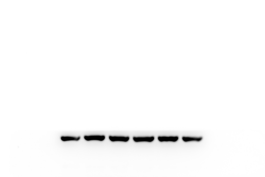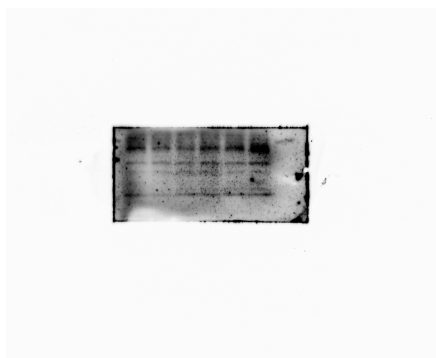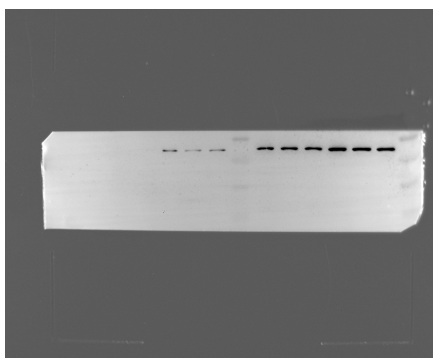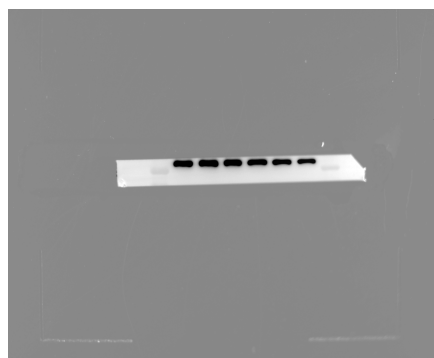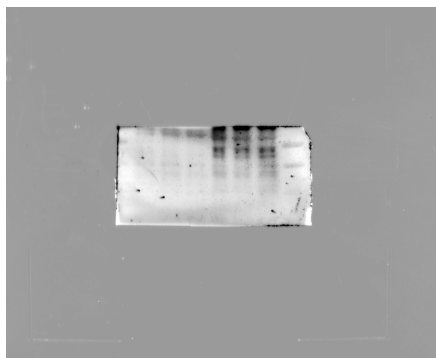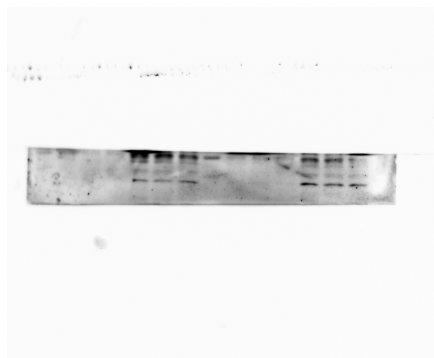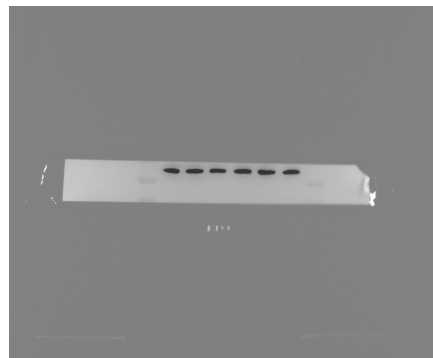

fig6

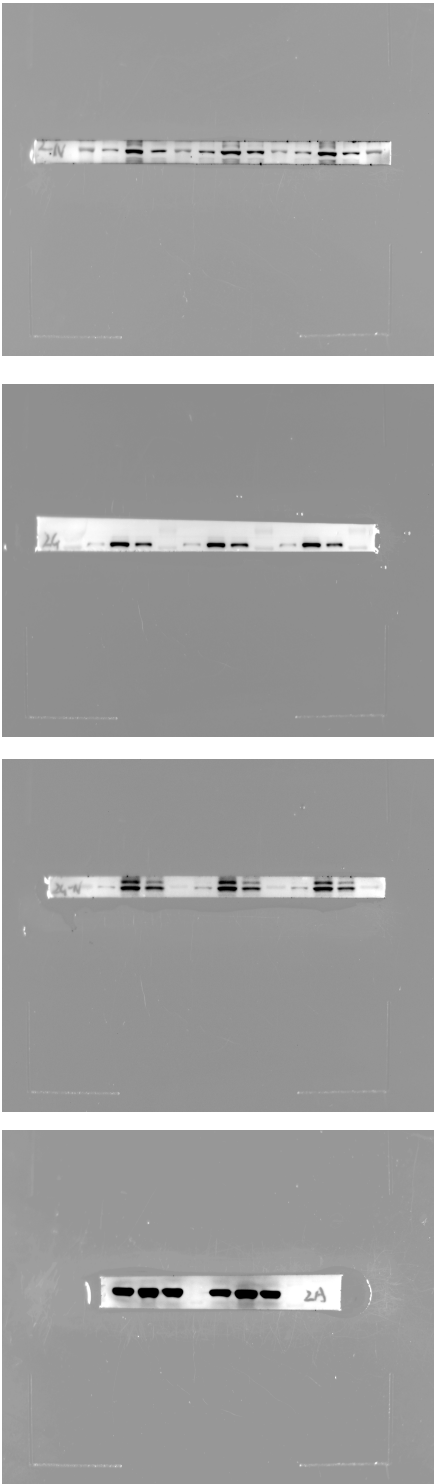

fig6

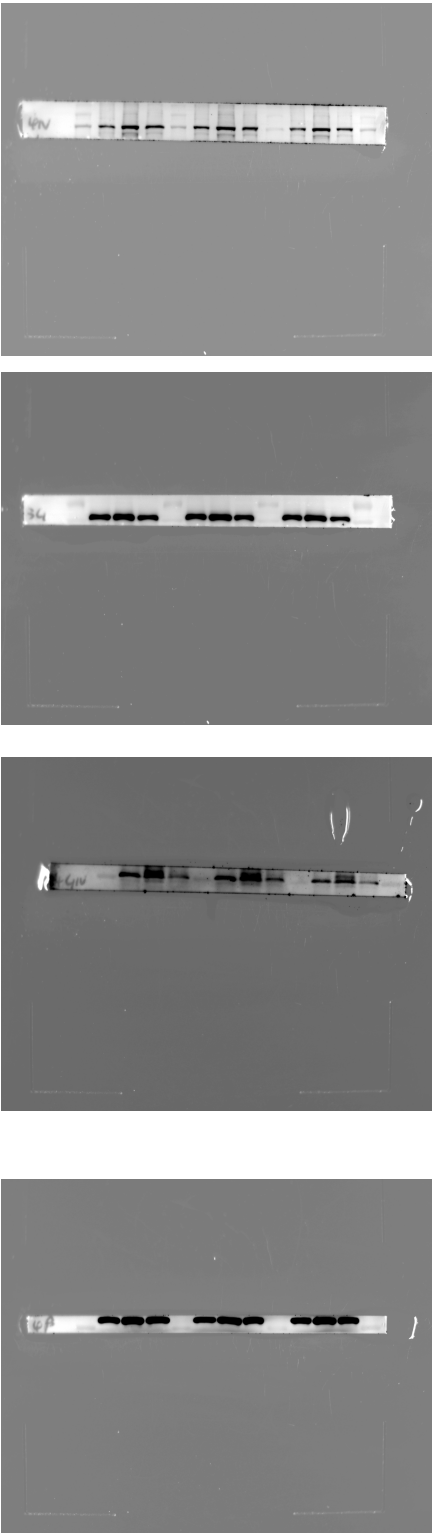

Supplement: Supplementary file 1 — Western blot raw bands [file 41420_2024_2105_MOESM1_ESM.pdf]
