# Supplementary material for: EZH2–STAT3 signaling pathway regulates GSDMD-mediated pyroptosis in glioblastoma
Source: Cell Death Discov. 2024 Jul 28;10:341. doi: 10.1038/s41420-024-02105-0 (PMC11284224; doi:10.1038/s41420-024-02105-0)

Figure 1 presents statistical analyses of wound healing and flow cytometry. The left panel illustrates wound healing analysis, while the right panel depicts flow cytometry results. Data are represented as mean ± standard deviation (SD), and statistical significance was determined using t-test.

Additionally, Western blot analyses were performed to assess NLRP3, STAT3, p-STAT3, GSDMD, N-GSDMD, IL-1β, IL-18 expression. The Western blot statistical analyses are summarized in figures of the supplementary material. Quantification is presented as mean ± SD, with statistical significance determined using t-test and ANOVA.


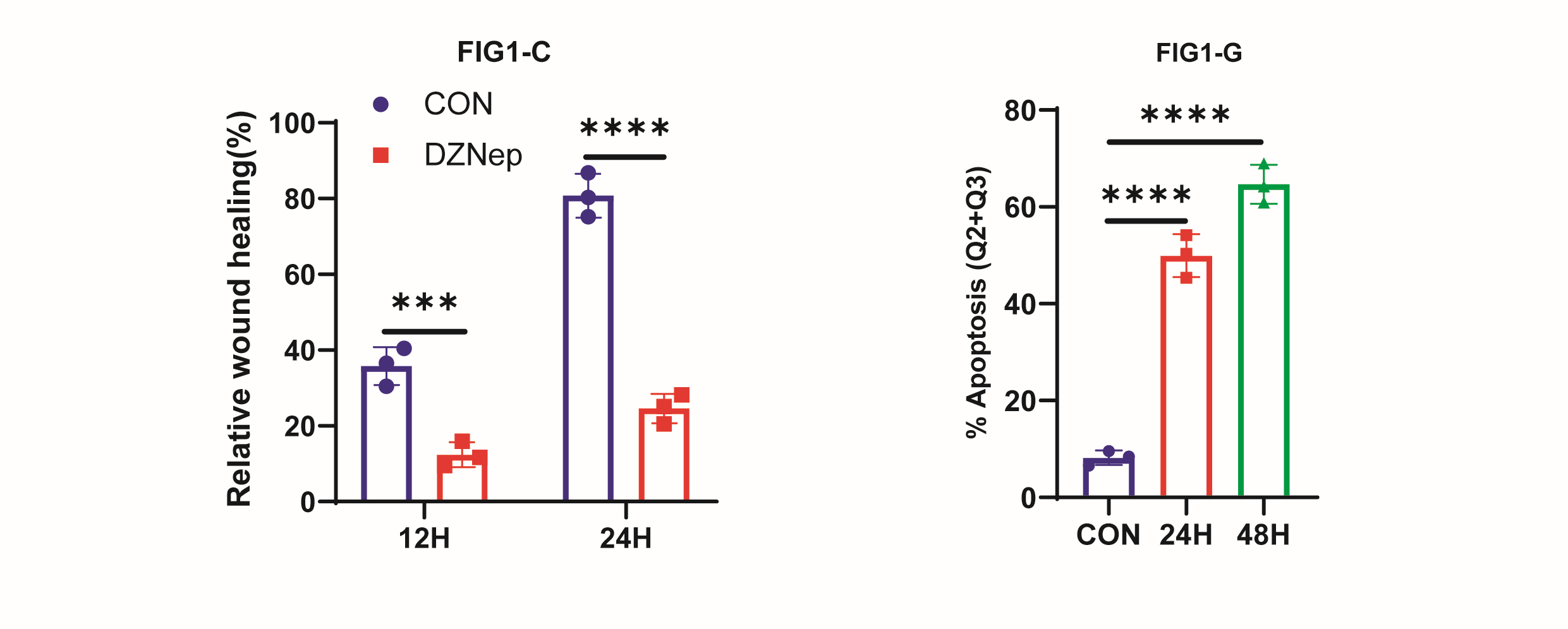


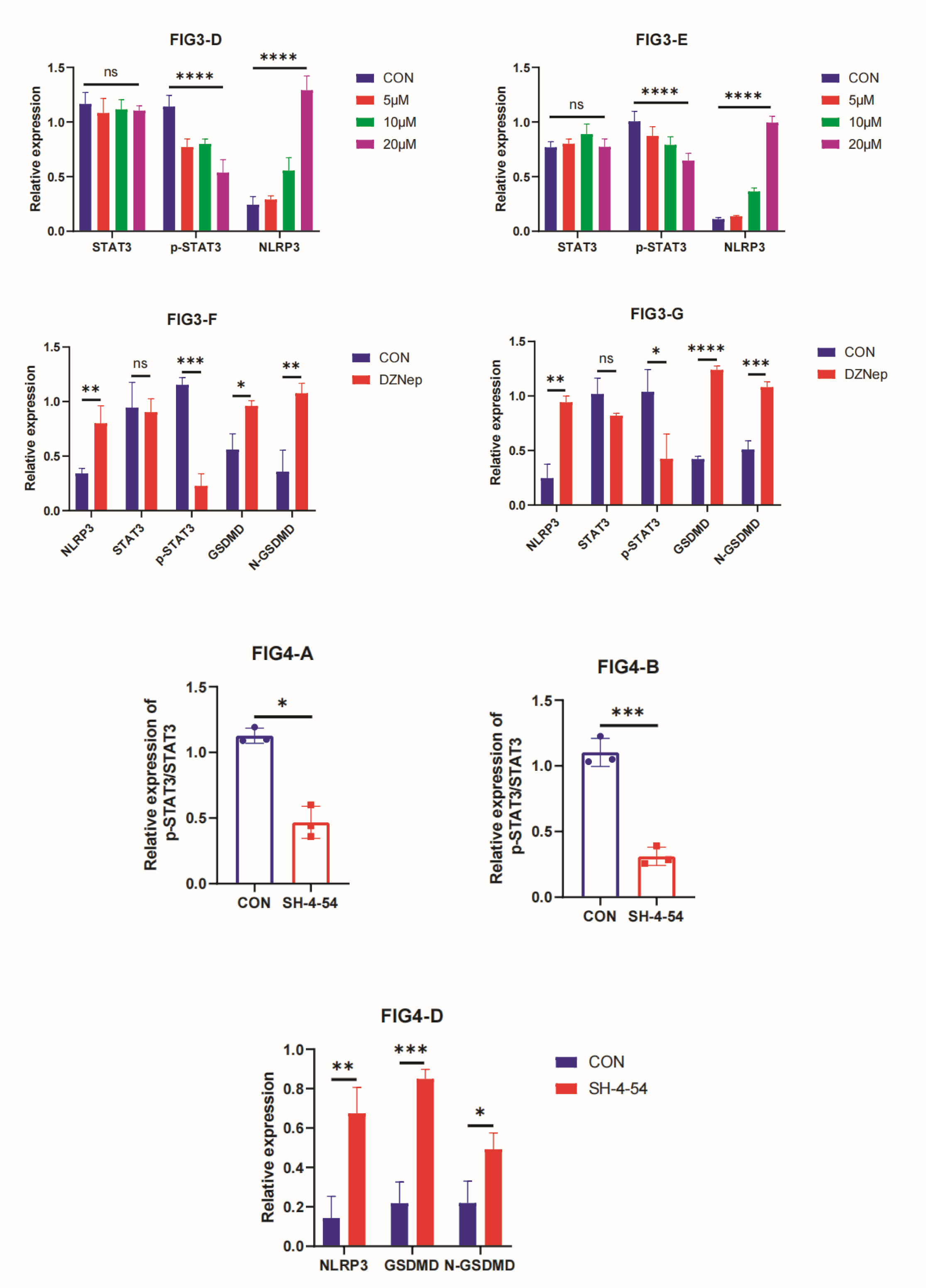

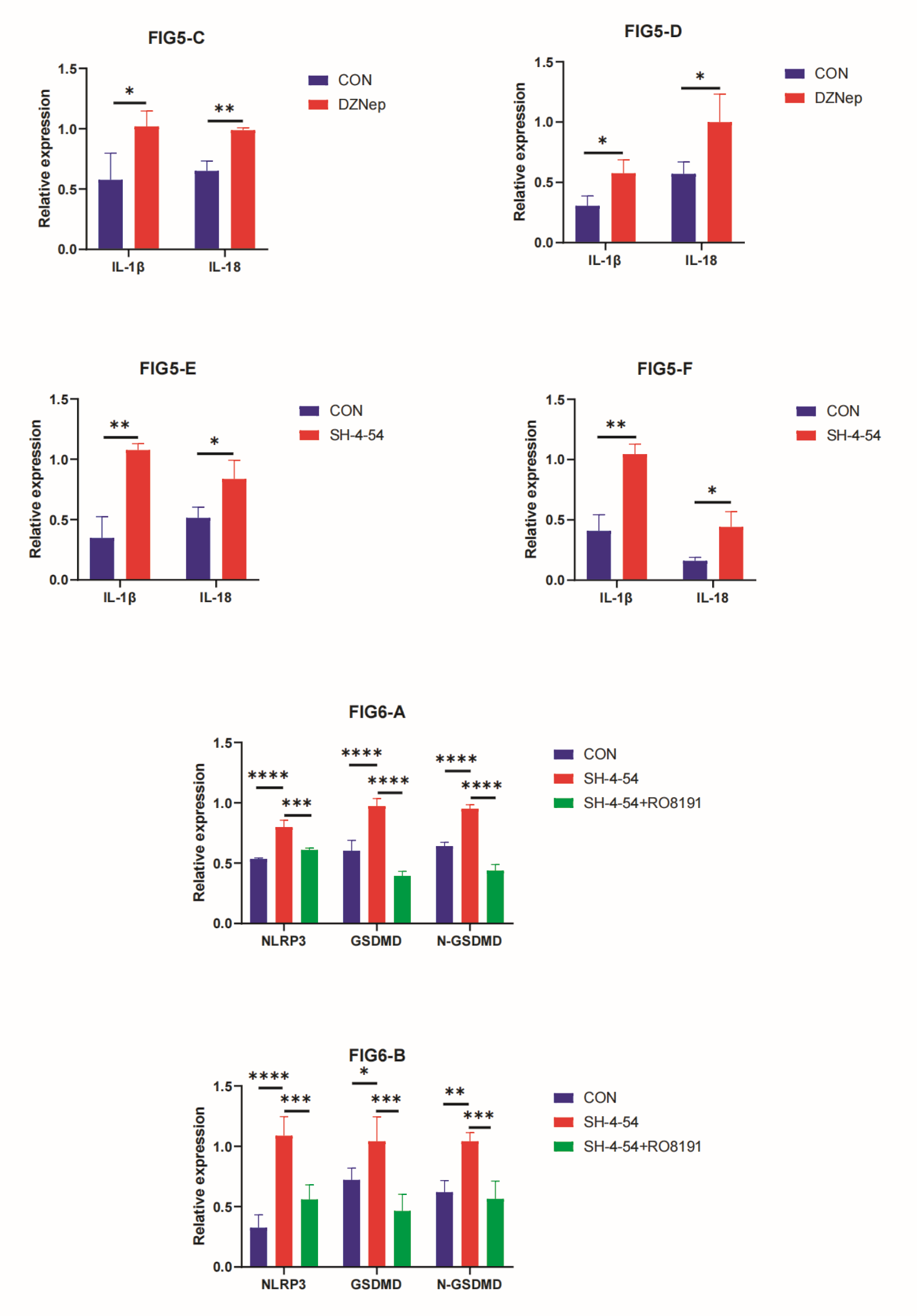

Supplement: Supplementary file 2 — Data statistics [file 41420_2024_2105_MOESM2_ESM.doc]
